# Dynamics of *phoD*- and *gcd*-Harboring Microbial Communities Across an Age Sequence of Biological Soil Crusts Under Sand-Fixation Plantation

**DOI:** 10.3389/fmicb.2022.831888

**Published:** 2022-03-04

**Authors:** Xingxing Zhao, Ying Zhang, Zhenbo Cui, Lu Peng, Chengyou Cao

**Affiliations:** College of Life and Health Sciences, Northeastern University, Shenyang, China

**Keywords:** soil crust, sand-fixation plantation, *phoD* gene, *gcd* gene, microbial diversity

## Abstract

Biological soil crusts (BSCs) are important for restoring vegetation and improving soil fertility in arid or semiarid desertified land. However, studies on the contribution of BSC microbes to phosphorus (P) transformation remains limited. The microbial diversity involved in P transformation and its dynamic along BSC development should be examined to further understand the microbial regulatory mechanism of the P-cycling process. This paper investigates the soil properties, P fractions, and potential of P transformation across a chronosequence (0-, 8-, 20-, and 35-year) of the BSC under *Caragana microphylla* plantation on the moving sand dunes in Horqin Grassland, China. An abundance of *phoD* and *gcd* genes was detected, and the diversities and structures of *phoD*- and *gcd*-haboring microbial communities were illustrated *via* high-throughput sequencing. Soil nutrient content, activity of alkaline phosphomonoesterase, potential of organic P (OP) mineralization, and the abundance of *phoD* and *gcd* genes all linearly increased along with BSC age. The microbial quantity and species diversity of the *phoD* community were greater than those of *gcd*. BSC development increased the availability of inorganic P (IP) fractions, and both NaHCO_3_-Pi and NaOH-Pi were positively correlated with the abundance of the two genes and the activity of alkaline phosphomonoesterase. The phyla of Actinobacteria, Planctomycetes, and Proteobacteria and the family of *Streptomycetaceae* were the most dominant taxa in the *phoD* community, Proteobacteria was the dominant phylum in the *gcd* community in BSC soils, and *Rhizobium* and *Planctomyces* were the most dominant genera. The dominant taxa quantitatively responded to soil property improvement, but the basic compositions and dominant taxa did not change along with BSC development. The structures of *phoD* and *gcd* communities were linked to soil properties, and pH available K, and total K tend to be the direct determining factors.

## Introduction

Revegetation *via* planting indigenous vascular plants on moving or semi-moving sand dunes has been widely adopted as an effective measure for controlling desertification. With the stabilization of moving sand dunes, biological soil crusts (BSCs) were subsequently formed on the soil surface. BSCs are assemblages of mosses, lichens, cyanobacteria, bacteria, and fungi that are commonly distributed on the soil surface in arid and semiarid regions throughout the world (Zhao et al., 2011). The development of BSC in these regions is affected mainly by vegetation cover, soil properties, and the interaction between organisms and soil (Hawkes, 2003). BSCs have important roles in desert ecosystems, such as in stabilizing the sand surface (Song et al., 2020), mitigating wind erosion (Zhao et al., 2011), facilitating water infiltration (Guan et al., 2019, 2021), enhancing soil fertility by fixing carbon and nitrogen, increasing the availability of nutrients for vascular plants (Zhang et al., 2014a; Morillas and Gallardo, 2015), and promoting the stabilization of sand dunes (Zhao et al., 2020a). In recent years, the nutrient cycling functions of microbes in BSCs have received much research attention (Zhang et al., 2014b; Morillas and Gallardo, 2015; Zhao et al., 2020b). Accordingly, many studies have investigated the diversities of microbial functional taxa and the functional genes of BSC involved in carbon and nitrogen cycles (Yeager et al., 2012; Abed et al., 2013; Pepe-Ranney et al., 2016; Liu et al., 2018; Zhao et al., 2020a). These studies provide useful information on the microbial mechanisms by which BSC regulate the forms and contents of soil carbon and nitrogen. However, studies on the contributions of BSC microbes to phosphorus (P) transformation remain limited. The microbial taxa related to P transformation and their dynamics along BSC development should be examined to further understand the microbial regulatory mechanisms of the P-cycling process.

Phosphorus is an essential macro-element, but its low availability often limits the growth and development of living organisms (Elser et al., 2007; Turner et al., 2015). Organisms depend on organic P (OP) mineralization and phosphate solubilization for their P nutrition and have developed complex adaptations to efficiently utilize scarce resources (Lambers et al., 2006; Turner et al., 2015). Soil microbes are determinants of the composition of P forms and key drivers of P transformation (Fraser et al., 2015; Hu et al., 2020). Alkaline phosphatase (ALP), which generally originates from soil microbes, is a key enzyme involved in hydrolyzing OP for planting potentially available orthophosphate in soil (Ragot et al., 2015; Turner et al., 2015; Romanyà et al., 2017). At least three homologous prokaryotic genes (*phoA*, *phoX*, and *phoD*) have been identified as encoding ALP based on their sequence similarity and substrate specificity (Luo et al., 2009; Kathuria and Martiny, 2011). These three genes are widely distributed in aquatic and terrestrial ecosystems, with the *phoD* gene being most frequently observed in terrestrial ecosystems (Luo et al., 2009; Tan et al., 2013; Dai et al., 2014). Therefore, the *phoD* gene can be treated as a key molecular marker for estimating the transformation of soil OP, and its abundance can be used to measure ALP bacterial diversity (Hu et al., 2018, 2020).

Phosphate-solubilizing bacteria (PSB) in soil can release soluble phosphate from insoluble mineral phosphate. During the growth and reproduction of PSB, various organic acids are secreted to facilitate the reduction of soil pH and dissolution of insoluble phosphate (Zeng et al., 2016; Li et al., 2019a). Gluconic acid (GA) is the most important dissolving phosphoric acid produced by PSB (Li et al., 2019b). Quinoprotein glucose dehydrogenase (encoded by the *gcd* gene), which catalyzes the oxidation reaction of glucose into GA, is considered the most fundamental enzyme in microbial P metabolism. Liang et al. (2020) performed sampling at a P-deficient degraded mine in Southern China and reconstructed 39 near-complete bacterial genomes harboring *gcd*. They found that the relative abundance of these genomes is significantly correlated with bioavailable soil P and confirmed that *gcd*-PSB has an important role in improving soil P cycling during the restoration of the degraded mine.

The objectives of this study are to (1) evaluate the P dynamics and differences in P speciation along an age sequence of BSC, (2) quantify the responses of *phoD* and *gcd* community compositions to the development of BSC, and (3) discuss the interactions of *phoD* and *gcd* communities with soil properties. Soil phosphatase activity and available soil P are hypothesized to increase along with BSC age possibly due to the altered structures of *phoD*- and *gcd*-harboring bacterial communities resulting from the improvement of soil nutrient status. Results of this work are expected to contribute novel knowledge on how P availability in degraded sandy soils can be enhanced by regulating *phoD*- and *gcd*-bacterial communities *via* soil amelioration.

## Materials and Methods

### Study Location and Site Description

This study was conducted at the Wulanaodu Experimental Station of Desertification Control (43° 02′ N, 119° 39′ E; altitude: 480 m) of the Chinese Academy of Sciences. The station is located in the western Horqin Sandy Land in Northeastern China, whose landscape is characterized by a mosaic of undulating sand dunes and interdune lowlands. The soils in this area are classified as cambic arenosols (FAO, 2006). The region lies within a temperate zone and has a continental semiarid monsoon climate. The original vegetation has been destroyed as a result of severe desertification in recent decades due to long-term overgrazing, excessive reclamation, and overcutting. In desertification control, *Caragana microphylla*, an indigenous leguminous shrub, has been widely considered the pioneer species for revegetation and moving sand fixation. A large area of *C. microphylla* plantation has been planted on the desertified sandy land around the Wulanaodu Region since the 1980s with the help of sand-protecting barriers (1 m × 1 m squares made of straw). The experimental site was enclosed after seeding, and BSCs gradually developed on the sand surface after several years. At present, an age sequence of *C. microphylla* plantation is well distributed in this region, with the oldest chronosequence being 35 years old as of the sampling time.

### Experimental Design and Soil Sampling

Soils were sampled in August 2019. Crust samples from 8-, 20-, and 35-year-old *C. microphylla* plantations (designated as BSC-8, BSC-20, and BSC-35, respectively) were collected. Three adjacent non-vegetated moving sand dunes (0-year, designated as MS) were also sampled as controls. Three sites of each plantation and MS were set up at different sand dune for sampling. Each site was located 200 m away from the other site. In each site, one plot (10 m × 10 m) was set up, and 15 subsamples were randomly collected and mixed into one sample. Half of each sample was immediately frozen at −80°C for DNA extraction, whereas the other half was stored in a 4°C refrigerator for enzymatic activity test and then air dried to analyze their chemical and physical properties.

### Soil Property Measurements

Soil moisture (SM) was measured using the drying method. Soil pH and electrical conductivity (EC) were measured in soil–water suspensions [1:2.5 and 1:5 soil–water ratios, respectively; Institute of Soil Science, Chinese Academy of Sciences (ISSCAS), 1978]. Soil organic matter (SOM) and total nitrogen (TN) were determined using the K_2_Cr_2_O_7_–H_2_SO_4_ oxidation and the semimicro-Kjeldahl digestion methods (Nelson and Sommers, 1982), respectively. Soil NH_4_-N was extracted using a 1 M KCl solution and determined using an automated discrete analyzer (CleverChem 380, Germany). Soil available P (AP) was determined using the Olsen and Dean method. Total potassium (TK) and available K (AK) were measured using atomic absorption spectroscopy [Institute of Soil Science, Chinese Academy of Sciences (ISSCAS), 1978]. ALP activity was determined using the methods described by Sardans and Peñuelas (2005).

Soil P fractions were determined using the sequential extraction method described by Hedley et al. (1982) with some modifications (da Silva et al., 2017). To determine H_2_O-Pi (P in water), 0.5 g soil was extracted by 30 ml distilled water and then centrifuged at 4°C 3,500 rpm for 16 min. The supernatant was separated by using a 0.45 μm filter membrane, and the inorganic P (IP) was measured. Fractioning was performed following the same logic as described in the previous steps with the following sequence of reagents: 0.5 mol L^−1^ NaHCO_3_ (NaHCO_3_-P), 0.1 mol L^−1^ NaOH (NaOH-P 0.1 mol L^−1^), 0.5 mol L^−1^ NaOH (NaOH-P 0.5 mol L^−1^), and 1 mol L^−1^ HCl (HCl-P). The residual soil was air dried and digested for 3 h at 190°C with HF and H_2_SO_4_ (5:3; residual-P). An aliquot of each extract was analyzed separately for TP. The organic P of each extract was independently calculated based on the difference between TP and IP. The general P (OP) from fractioning was computed as the sum of P from all fractions (da Silva et al., 2017).

The potential of P transformation of soil microbes was determined using the culture method proposed by Zheng and Zhang (1982). Specifically, 10 g fresh soil was weighed into a triangular flask. Afterward, 50 ml sterile water was added and shaken for 30 min using a shaker. The basic microbial culture medium comprised 0.5 g (NH_4_)_2_SO_4_, 0.3 g MgSO_4_.7H_2_O, 5.0 g CaCO_3_, 0.3 g KCl, 0.3 g NaCl, minute amounts of MnSO_4_ and FeSO_4_, 100 g glucose, and 1,000 ml distilled water. About 35 ml culture mediums and 35 mg lecithin or 35 mg phosphorite were then added in a conical flask, and high-pressure steam sterilization was performed. Afterward, a 10 ml soil–water suspension was inoculated in the sterilized culture medium at 30°C for 21 days in darkness. After incubation, the AP in the culture mediums was measured. The proportions of AP to total P in lecithin and phosphorite-added medium were then estimated to denote the potential mineralization abilities of OP and the dissolution of IP, respectively.

### Soil DNA Extraction, Real-Time PCR, and Sequencing of *phoD* and *gcd* Genes

The soil genomic DNA from MS, BSC-8, BSC-20, and BSC-35 sites was extracted using the Soil DNA Quick Extraction Kit (Bioteke, China). The fragment of the *phoD* gene was amplified by PCR with the primer pair F1-TGGGAYGATCAYGARGT and R1-CTGSGCSAKSACRTTCCA (Ragot et al., 2015), and *gcd* was amplified by PCR with the primer pair F2-CGGCGTCATCCGGGSITIYRAYRT and R2-GGGCATGTCCATGTCC (Bergkemper et al., 2016). The purified PCR products were sequenced on an Illumina MiSeq platform (Shanghai Personal Biotechnology Co., Ltd., Shanghai, China). The operational taxonomic units (OTUs) with 97% similarity cutoff were clustered. The representative sequences of each OTU were taxonomically classified using the BLAST algorithm-based search within GenBank.^1^ OTU richness analysis was conducted using the Mothur software (version 1.21.1),^2^ and the alpha diversity indices, including Chao’s species richness estimator (Chao 1), Shannon–Wiener index, Pielou index, and Simpson diversity index, were calculated (Schloss et al., 2009). Hierarchical clustering analysis was performed using the unweighted pair-group method with arithmetic means (UPGMA) to contrast the community structures of different samples, and a pairwise analysis of similarity (ANOSIM) cross the four sample types was performed to test the significance of the difference. The distinctive taxa in different sites were identified using the linear discriminant analysis (LDA) effect size (LEfSe) method (Segata et al., 2011), and the Pearson correlations between the distinctive taxa and soil properties were calculated. All *phoD* and *gcd* gene sequences were submitted to the NCBI Sequence Read Archive under accession number SRP190783.

The *phoD* and *gcd* genes were quantified through real-time quantitative PCR (RT-qPCR) by using a Q5 Real-Time PCR System (Applied Biosystems, United States) with SYBR Green as the fluorescent dye in a 20 μl reaction mixture containing 1.0 μl of each primer for *phoD* or *gcd*, 1.0 μl DNA template, 2.5 μl BSA, and 10 μl Gotaq q-PCR Master Mix (Promega, France). The reaction programs: 95°C for 5 min, followed by 35 cycles of 94°C for 30 s, 57°C for 1 min, 72°C for 1 min, and a final extension of 10 min at 72°C. Two independent real-time PCR assays were then conducted for the two genes and each soil replicate.

### Data Analysis

The responses of soil physicochemical properties, ALP activity, soil P fractions, P transformation rate, and copies of *phoD* and *gcd* genes to BSC age were evaluated using the linear regression model. The bivariate correlations were performed and pairwise independent Pearson correlations among soil P fractions, P transformation rate, and abundance of *phoD* and *gcd* genes were calculated. All statistical analyses were performed using SPSS version 18.0, and statistical significance was set to *p* ≤ 0.05. Redundancy analysis (RDA) was performed using CANOCO 4.5 to identify which soil factors exhibit the most significant effects on the variations in dominant *phoD* and *gcd* taxa, and the correlations of the soil parameters were examined by a Monte Carlo permutation.

## Results

### Soil Properties at the Time of Sampling

Results of the determinations of SM, pH, EC, SOM, TN, TK, NH_4_-N, and AK of the age sequence of BSCs are shown in Table 1. Significant differences were observed in the abovementioned indictors across different BSC sites. The SOM, TN, NH_4_-N, AK, and EC in BSC-8, BSC-20, and BSC-35 sites were 2.73–5.31, 8.90–10.25, 6.89–10.30, 2.76–3.63, and 1.21–1.48 times larger than those in MS sites, respectively. Similarly, the activities of soil ALP were 54.03–131.08 times larger, respectively. These results suggest that BSC formation has a more significant improvement effect on soil ALP activity than on soil nutrients. Results of the regression analysis revealed that soil pH, EC, nutrients (except for TK), and ALP had significantly linear relationships with BSC age (*p* ≤ 0.05; Table 1), hence indicating their increasing tendencies to BSC development.

**Table 1:** Soil properties in an age sequence of soil crusts under plantations.

	MS	BSC-8	BSC-20	BSC-35	ANOVA in response to plantation age			
					Regress equation	*R* ^2^	*F*	*P*
Soil moisture (%)	0.273 ± 0.165	0.600 ± 0.061	0.507 ± 0.085	0.580 ± 0.0721	-	0.283	3.952	0.075
pH	5.587 ± 0.101	6.843 ± 0.006	7.543 ± 0.032	7.003 ± 0.160	*y* = 0.037*x* + 6.16	0.461	8.552	0.015
EC (μs cm^−1^)	45.20 ± 4.464	99.87 ± 7.253	101.43 ± 5.720	112.1 ± 13.66	*y* = 1.584*x* + 64.707	0.599	14.939	0.003
SOM (%)	0.160 ± 0.062	0.597 ± 0.098	0.867 ± 0.040	1.010 ± 0.035	*y* = 0.023*x* + 0.297	0.852	57.689	<0.001
Total N (%)	0.002 ± 0.0019	0.0198 ± 0.001	0.0221 ± 0.003	0.0225 ± 0.001	*y* = 0.0001*x* + 0.0029	0.566	13.068	0.005
Total K (g kg^−1^)	28.04 ± 4.736	24.31 ± 0.995	25.58 ± 0.668	26.22 ± 0.705	-	0.017	0.170	0.688
NH_4_-N (mg kg^−1^)	1.588 ± 0.145	13.54 ± 0.730	17.95 ± 2.857	12.512 ± 1.019	*y* = 0.269*x* + 7.163	0.332	4.974	0.050
Available K (mg kg^−1^)	46.24 ± 2.960	174.0 ± 14.91	176.5 ± 14.59	214.0 ± 12.07	*y* = 4.008*x* + 89.556	0.678	21.077	0.001
ALP (mg g^−1^ h-^1^)	3.089 ± 1.176	170.0 ± 35.95	241.9 ± 85.79	408.0 ± 21.61	*y* = 10.877*x* + 32.024	0.940	94.287	<0.001

Values are means ± SD. MS, moving sand dune (0-year); BSC-8, 8-year soil crust; BSC-20, 20-year soil crust; BSC-35, 35-year soil crust; EC, electrical conductivity; SOM, soil organic matter; and ALP, alkaline phosphomonoesterase.

### P Fractions and Potential of P Transformation

The available IP fractions (H_2_O-Pi and NaHCO_3_-Pi) increased along with BSC age, but their values were the lowest among all P fractions (Table 2). The concentrations of the AP stock (H_2_O-Pi+NaHCO_3_-Pi+NaHCO_3_-Po) tended to increase along with BSC age, and the values in BSC-8, BSC-20, and BSC-35 sites were 27.51, 26.12, and 33.95% higher than those in the MS site, respectively. The most abundant fraction was the OP extracted by NaOH and HCl, which accounted for 55.69% of the general P. Each fraction in the MS site (except for 0.5 mol L^−1^ NaOH-Po) was lower than those in the BSC sites. Results of the regression analysis suggested that the contents of 0.5 mol L^−1^ NaHCO_3_-Pi, 0.1 mol L^−1^ NaOH-Pi, 1.0 mol L^−1^ HCl-Po, 0.5 mol L^−1^ NaOH-Pi, residual-P, and general P significantly and linearly increased along with BSC age (*p* < 0.05; Table 2). Significant correlations were also found among NaHCO_3_-Pi (0.5 mol L^−1^), NaOH-Pi (0.1 mol L^−1^), and ALP (*p* < 0.01; Table 3).

**Table 2:** Fractions of soil P in an age sequence of soil crusts under plantations (mg kg^−1^).

	MS	BSC-8	BSC-20	BSC-35	ANOVA in response to plantation age			
					Regress equation	*R* ^2^	*F*	*P*
H_2_O-Pi	2.585 ± 0.169	0.805 ± 0.150	2.920 ± 0.540	2.835 ± 0.678	-	0.164	1.955	0.192
NaHCO_3_-Pi (0.5 mol L^−1^)	3.870 ± 0.098	7.010 ± 1.129	10.35 ± 1.043	12.51 ± 2.645	*y* = 0.243*x* + 4.605	0.835	50.715	<0.001
NaHCO_3_-Po (0.5 mol L^−1^)	56.13 ± 7.969	71.99 ± 10.23	65.66 ± 5.505	68.49 ± 2.115	-	0.127	1.449	0.256
NaOH-Pi (0.1 mol L^−1^)	4.435 ± 0.369	8.010 ± 0.781	12.76 ± 1.783	12.80 ± 0.717	*y* = 0.242*x* + 5.688	0.780	35.507	<0.001
NaOH-Po (0.1 mol L^−1^)	72.57 ± 1.559	82.99 ± 27.09	92.24 ± 18.03	83.20 ± 20.47	-	0.052	0.553	0.474
HCl-Pi (1.0 mol L^−1^)	3.990 ± 0.234	19.56 ± 0.788	16.42 ± 1.157	16.65 ± 2.307	-	0.298	4.246	0.066
HCl-Po (1.0 mol L^−1^)	94.01 ± 4.809	97.44 ± 3.471	112.6 ± 2.263	105.6 ± 3.608	*y* = 0.389*x* + 96.226	0.433	7.640	0.020
NaOH-Pi (0.5 mol L^−1^)	4.200 ± 0.398	8.695 ± 0.345	9.160 ± 1.435	9.290 ± 0.162	*y* = 0.121*x* + 5.932	0.525	11.043	0.008
NaOH-Po (0.5 mol L^−1^)	78.80 ± 9.563	66.31 ± 9.236	83.84 ± 8.069	81.71 ± 22.64	-	0.063	0.677	0.430
Residual-P	84.33 ± 0.389	86.90 ± 6.301	85.10 ± 1.126	94.62 ± 5.732	*y* = 0.260*x* + 83.639	0.407	6.868	0.026
General-P	404.9 ± 13.78	457.7 ± 29.99	511.0 ± 15.95	522.5 ± 22.47	*y* = 3.307*x* + 421.934	0.763	32.193	<0.001

Values are means ± SD. MS, moving sand dune (0-year); BSC-8, 8-year soil crust; BSC-20, 20-year soil crust; and BSC-35, 35-year soil crust.

**Table 3:** Correlation coefficients among fractions of soil P, *phoD*, and *gcd* gene abundance, alkaline phosphomonoesterase activity, and mineralization rate of lecithin in an age sequence of soil crusts under plantations (*n* = 12).

Item	NaHCO_3_-Pi (0.5 mol L^−1^)	NaOH-Pi (0.1 mol L^−1^)	ALP	MRL	PGC	GGC
NaHCO_3_-Pi (0.5 mol L^−1^)	1					
NaOH-Pi (0.1 mol L^−1^)	0.929^**^	1				
ALP	0.920^**^	0.913^**^	1			
MRL	0.769^**^	0.811^**^	0.787^**^	1		
PGC	0.840^**^	0.745^**^	0.861^**^	0.804^**^	1	
GGC	0.950^**^	0.839^**^	0.853^**^	0.770^**^	0.923^**^	1

ALP, alkaline phosphomonoesterase; MRL, mineralization rate of Lecithin; PGC, *phoD* gene copies; and GGC, *gcd* gene copies.

** *p* < 0.01.

**Figure 1:**
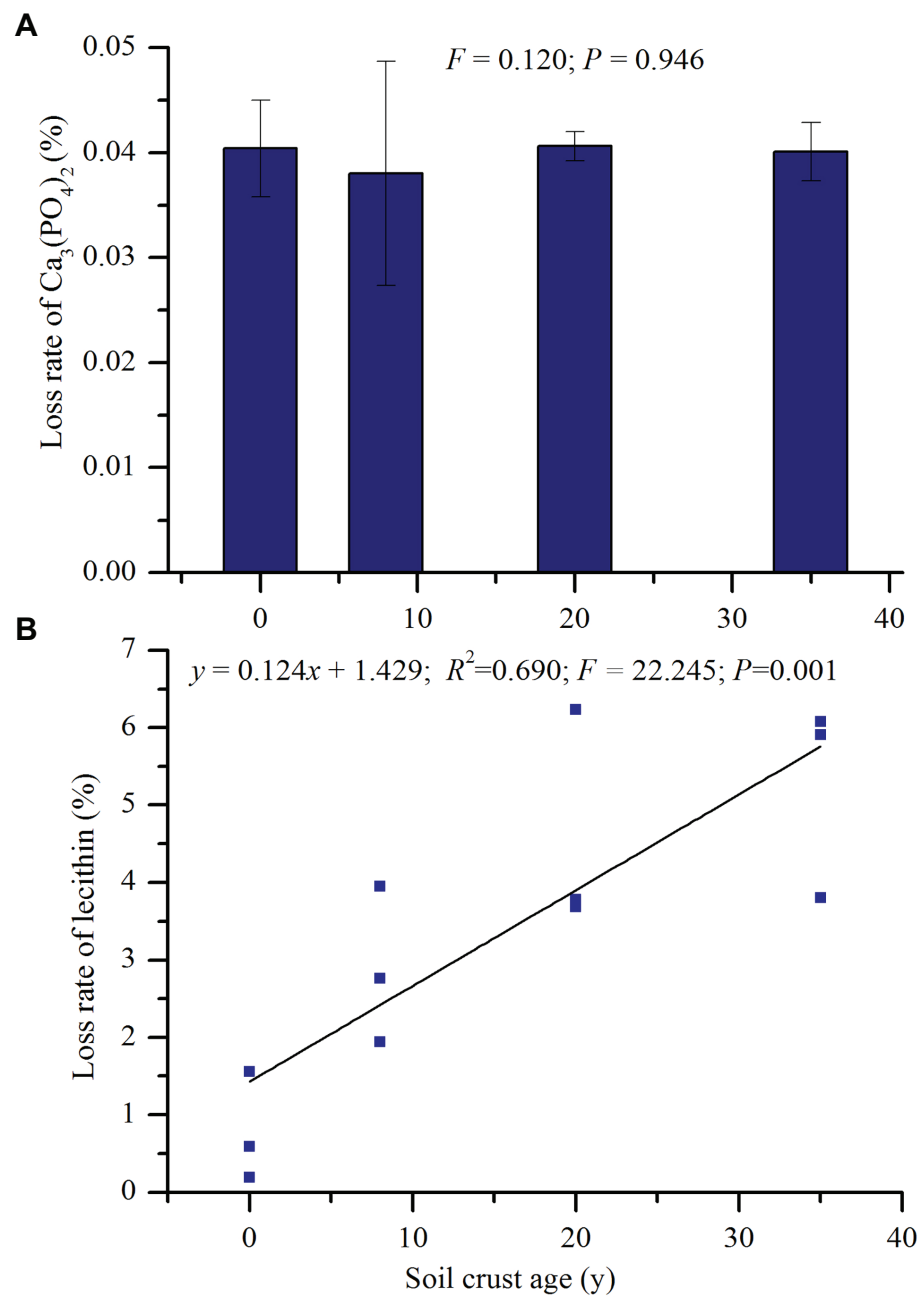
Responses of the potentials of inorganic P (IP) solubilization **(A)** estimated by the loss rate of phosphorite and organic P (OP) mineralization **(B)** estimated by the loss rate of lecithin quantitatively added in a microbial medium to biological soil crust (BSC) development.

The rates of phosphorite dissolution and lecithin mineralization were determined after 21 days incubation, and the results are presented in Figure 1. The average phosphorite dissolution rate was only 0.04%, and no significant difference was observed across different sites. Meanwhile, the lecithin mineralization rate ranged from 0.78% at the MS site to 5.27% at the BSC-35 site and linearly increased along with BSC age (*p* < 0.01). The correlation coefficients among the mineralization rates of lecithin, NaHCO_3_-Pi, and NaOH-Pi were all significant (*p* < 0.01; Table 3).

### Abundance of *phoD* and *gcd* Genes

**Figure 2:**
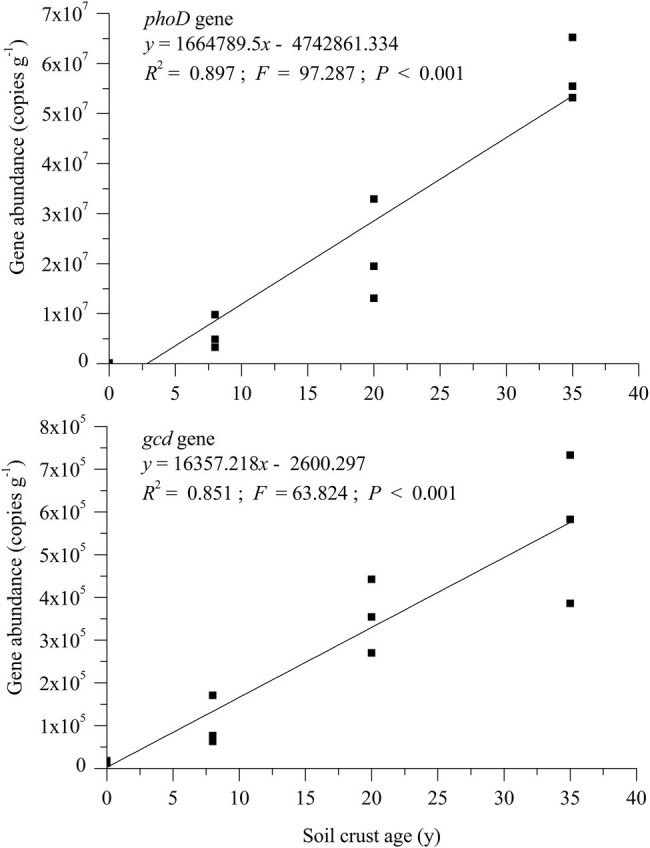
Abundance of *phoD* and *gcd* genes and their responses to BSC development.

Quantitative PCR was performed to measure the abundance of *phoD* and *gcd* genes in different soil samples. The number of *phoD* gene copies (PGC)/g dry soil ranged from 1.33 × 10^5^ to 5.80 × 10^7^, whereas that of the *gcd* gene ranged from 1.46 × 10^4^ to 5.67 × 10^5^ (Figure 2). The formation of BSC significantly facilitated an increase in abundance of genes *phoD* and *gcd*. The values of *phoD* and *gcd* in the BSC-8, BSC-20, and BSC-35 sites were 45.05–435.91 and 136.50–406.30 times larger than those in the MS site, respectively. Linear regression relationships were observed between the abundance of the two genes and plantation age (Figure 2; *p* < 0.001). Overall, the copies of *phoD* and *gcd* genes consistently increased with BSC age, and this tendency is similar to the variations in soil nutrients. The abundance of *phoD* and *gcd* genes was positively correlated with NaHCO_3_-Pi, NaOH-Pi (0.1 mol L^−1^), ALP activity, and lecithin mineralization rate (*p* < 0.05; Table 3).

### Compositions of *phoD* and *gcd* Communities

A total of 529,590 and 1,340,581 high-quality partial *phoD* and *gcd* gene sequences were obtained, respectively, for 12 samples after the quality trimming and removal of chimeras. All rarefaction curves tended to approach a plateau at the sequencing depth (Supplementary Figure S1), thereby suggesting that the volume of the sequenced reads was reasonable. The alpha diversity indices (including Shannon–Wiener, Simpson, Pielou, Chao 1, and observed species) were calculated (Table 4). The alpha diversity of the *phoD* community was much higher than that of *gcd*. The diversity indices of *phoD* and *gcd* communities (except for the Simpson of *gcd* and observed species) linearly increased along with BSC age (*p* < 0.05). The observed species in BSC samples were all significantly higher than those observed in MS, and the highest *phoD* and *gcd* values were observed in BSC-20 sites. Hierarchical clustering analysis divided the 12 samples into four groups and revealed significant differences in the *phoD* or *gcd* community structures across BSC development (Supplementary Figures S2, S3). The results of ANOSIM (*R* = 0.985, *p* < 0.01) also indicated the significant difference cross the four sample types.

**Table 4:** Diversity indices of *phoD* and *gcd* microbial communities in an age sequence of soil crusts.

Index		MS	BSC-8	BSC-20	BSC-35	ANOVA in response to soil crust age		
						*R^2^*	*F*	*P*
Chao1	*phoD*	830.6 ± 73.50	3317.3 ± 621.4	3634.6 ± 346.8	3038.9 ± 412.9	0.333	5.003	0.049
	*gcd*	845.8 ± 234.0	1384.7 ± 223.1	1626.8 ± 165.5	1181.9 ± 163.0	0.104	1.165	0.306
Shannon–Wienner	*phoD*	5.900 ± 0.168	9.538 ± 0.446	9.685 ± 0.257	9.053 ± 0.480	0.343	5.214	0.046
	*gcd*	3.074 ± 1.741	6.529 ± 0.333	6.835 ± 0.381	6.503 ± 0.525	0.368	5.828	0.039
Simpson	*phoD*	0.891 ± 0.009	0.996 ± 0.002	0.995 ± 0.003	0.992 ± 0.004	0.443	7.954	0.018
	*gcd*	0.604 ± 0.292	0.954 ± 0.007	0.961 ± 0.016	0.967 ± 0.005	0.316	4.622	0.057
Pielou	*phoD*	0.640 ± 0.012	0.886 ± 0.024	0.889 ± 0.015	0.853 ± 0.032	0.346	5.279	0.044
	*gcd*	0.350 ± 0.192	0.647 ± 0.024	0.663 ± 0.055	0.669 ± 0.060	0.378	6.075	0.033
Observed species	*phoD*	595.1 ± 40.83	1755.3 ± 256.7	1907.8 ± 166.4	1574.9 ± 184.0	0.302	4.322	0.064
	*gcd*	428.0 ± 103.9	1100.4 ± 177.0	1258.8 ± 163.0	851.9 ± 194.7	0.130	1.490	0.250

MS, moving sand dune (0-year); BSC-8, 8-year soil crust; BSC-20, 20-year soil crust; and BSC-35, 35-year soil crust.

**Figure 3:**
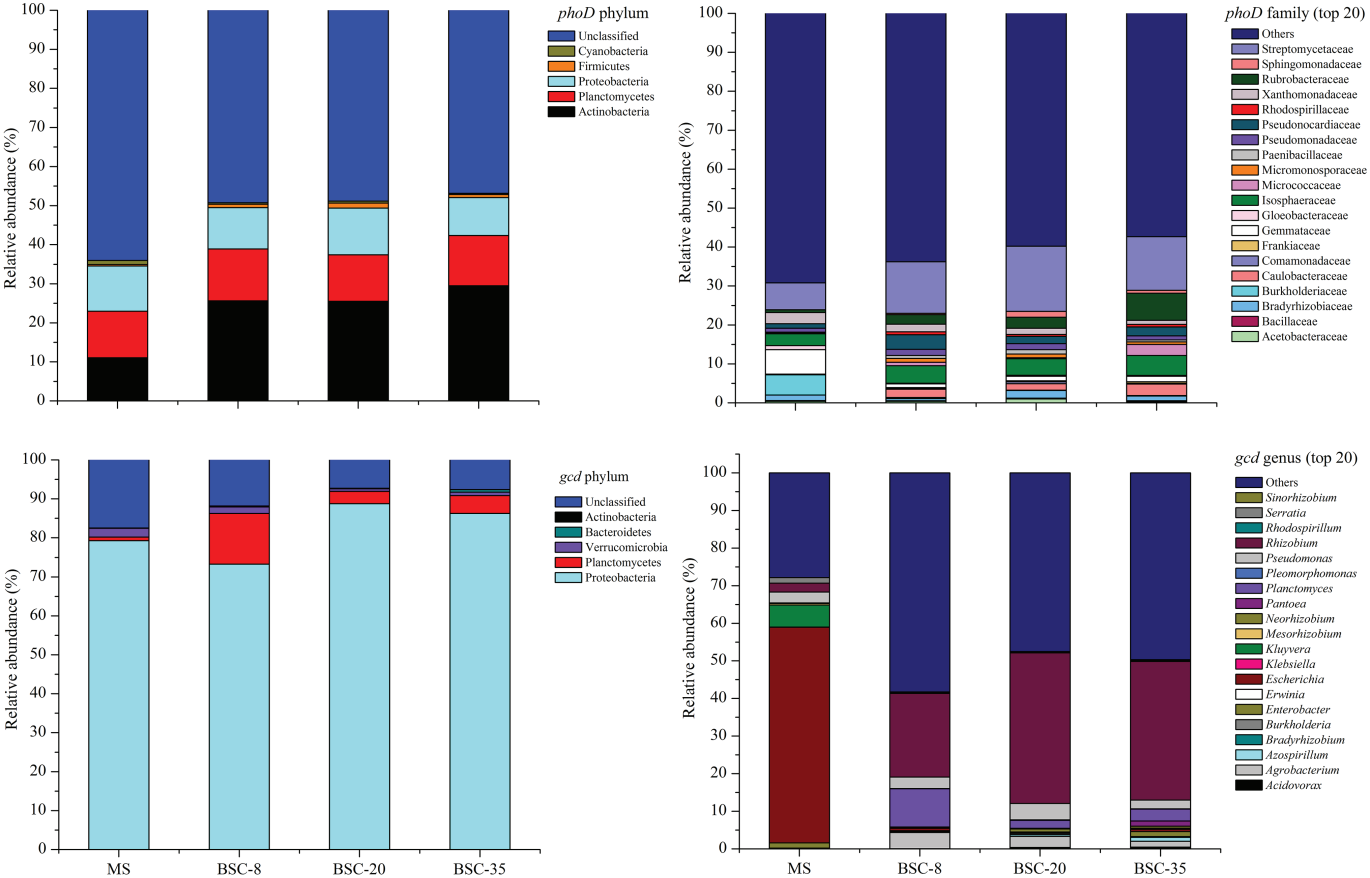
Relative abundance of the dominant taxa of *phoD* and *gcd* communities (average of three sites) across different sites. MS, moving sand dune; BSC-8, 8-year-old biological soil crust; BSC-20, 20-year-old biological soil crust; and BSC-35, 35-year-old biological soil crust.

All detected *phoD* OTUs were classified into five phyla, 22 orders, or 28 families. The detected *phoD* phyla included Actinobacteria (22.92%), Planctomycetes (12.49%), Proteobacteria (10.94%), Firmicutes (0.85%), and Cyanobacteria (0.54%). Meanwhile, the obtained *gcd* OTUs were classified into five phyla, 11 orders, 20 families, or 20 genera. The *gcd* phyla included Proteobacteria (81.91%), Planctomycetes (5.42%), Verrucomicrobia (1.37%), Bacteroidetes (0.25%), and Acidobacteria (0.001%; Figure 3). A total of 14 dominant *phoD* families and 10 *gcd* dominant genera with a relative abundance of ≥1% were observed. Among the dominant *phoD* families, Streptomycetaceae was the most dominant taxon in all samples with an average relative abundance of 12.65%, followed by Isosphaeraceae (4.24%), Rubrobacteraceae (3.22%), Gemmataceae (2.45%), and Pseudonocardiaceae (2.27%). Meanwhile, *Rhizobium* was the most abundant *gcd* genus in the BSC sites with an average relative abundance of 33.07%, followed by *Planctomyces* (5.107%), *Pseudomonas* (3.29%), and *Agrobacterium* (2.93%), whereas *Escherichia* was the absolute abundant genus in the MS site with an average relative abundance of 57.35%, followed by *Kluyvera* (5.88%), *Pseudomonas* (2.89%), and *Rhizobium* (2.35%; Figure 3).

**Figure 4:**
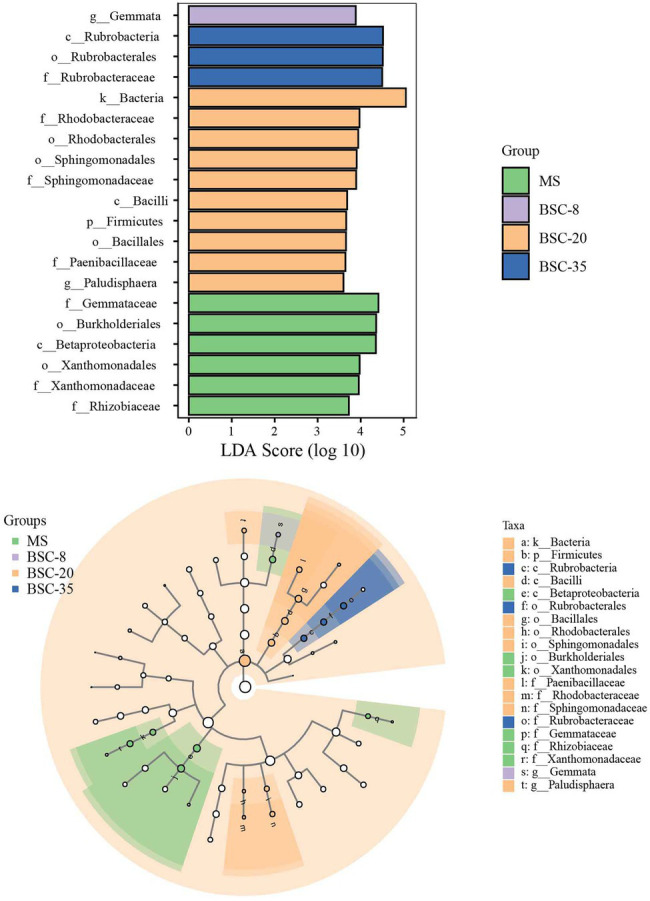
The linear discriminant analysis (LDA) effect size (LEfSe) method identifies the significantly different abundant taxa of the *phoD* community across different sites. Only the taxa with average abundances of >1% were considered significant. A significance level of 0.05 and an effect size threshold of 3 were used for all evaluated biomarkers. MS, moving sand dune; BSC-8, 8-year-old biological soil crust; BSC-20, 20-year-old biological soil crust; and BSC-35, 35-year-old biological soil crust.

**Figure 5:**
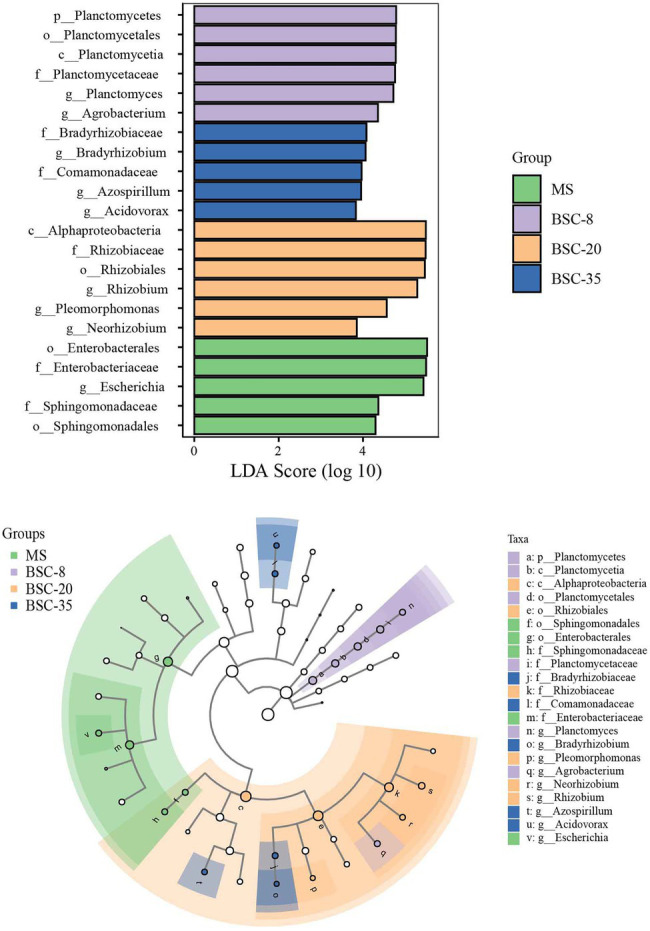
The LEfSe method identifies the significantly different abundant taxa of the *gcd* community across different sites. Only the taxa with average abundances of >1% were considered significant. A significance level of 0.05 and an effect size threshold of 3 were used for all evaluated biomarkers. MS, moving sand dune; BSC-8, 8-year-old biological soil crust; BSC-20, 20-year-old biological soil crust; and BSC-35, 35-year-old biological soil crust.

The LEfSe method was used to further analyze the differences in the relative abundance of *phoD* or *gcd* taxa across different sites, and the significantly different taxa in each site and their evolutionary relationships are shown in Figures 4, 5. In the MS site, the significantly different *phoD* taxa included members from Gemmataceae, Burkholderiales, Betaproteobacteria, Xanthomonadaceae, and Rhizobiaceae, and the marked *gcd* taxa included Enterobacterales, Enterobacteriaceae, *Escherichia*, and Sphingomonadaceae. In the BSC-8 site, *Gemmata* was the only distinctive *phoD* taxon, whereas Planctomycetales, *Planctomyces*, *Planctomycetes*, and *Agrobacterium* were the key *gcd* taxa. The distinctive *phoD* taxa in BSC-20 sites included *Bacteria*, Rhodobacteraceae, Sphingomonadaceae, *Bacilli*, Firmicutes, Paenibacillaceae, and *Paludisphaera*, whereas the marked *gcd* taxa included Alphaproteobacteria, *Rhizobium*, *Pleomorphomonas*, and *Neorhizobium*. The marked *phoD* taxon in the BSC-35 site was Rubrobacteraceae, whereas the marked *gcd* taxa included *Bradyrhizobium*, Comamonadaceae, *Azospirillum*, and *Acidovorax*. These results highlight the waxing and waning responses of the compositions of *phoD* and *gcd* communities to BSC development. Pearson correlations between the distinctive taxa (relative abundances > 1% in at least one sample) and soil properties were calculated and results were listed in Supplementary Table S1. In *phoD* community, the relative abundance of Burkholderiales, Betaproteobacteria, and Xanthomonadaceae significantly negatively correlated to most soil properties, while that of Sphingomonadaceae and Rubrobacteraceae significantly positively correlated to soil properties. Similarly, in *gcd* community, significantly negative relationships between some taxa (including Enterobacterales, Enterobacteriaceae, *Escherichia*, and Sphingomonadaceae) and soil properties, and significantly positive relationships between several taxa (*Agrobacterium*, Alphaproteobacteria, and *Rhizobium*) and soil properties were also observed. However, the relative abundance of Gemmataceae, Firmicutes, and Paenibacillaceae in *phoD* community and Planctomycetales, Planctomyces, and Planctomycetes in *gcd* community did not significantly correlated to soil properties.

### Dependence of *phoD* and *gcd* Communities on Environmental Variables

**Figure 6:**
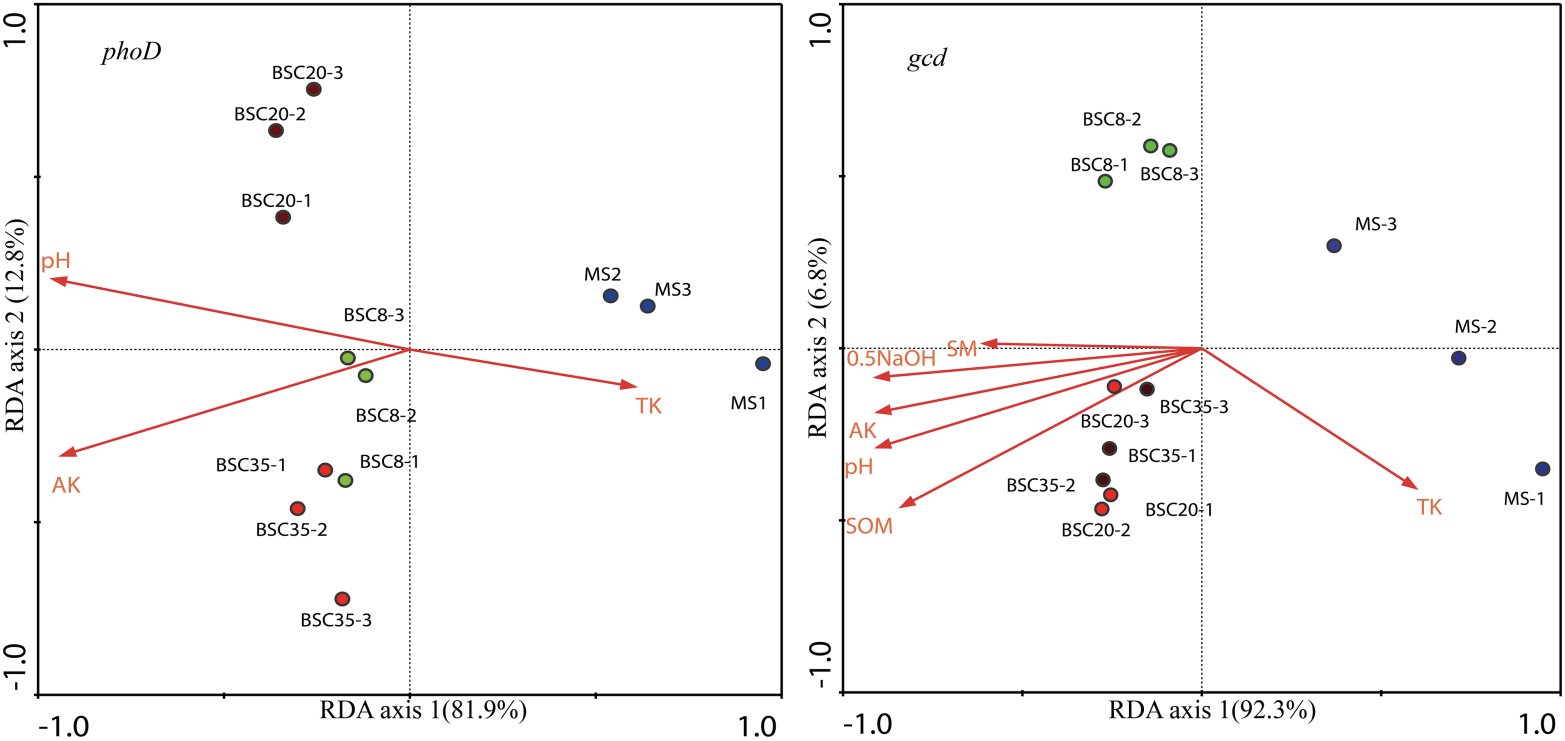
Redundancy analysis (RDA) of the structure of the *phoD* or *gcd* community and soil properties. SOM, soil organic matter; TK, total K; AK, available K; SM, soil moisture; 0.5 NaOH, 0.5 M NaOH-Pi; MS, moving sand dune; BSC-8, 8-year-old biological soil crust; BSC-20, 20-year-old biological soil crust; and BSC-35, 35-year-old biological soil crust.

Redundancy analysis was performed to analyze the relationships of the OTU relative abundance of dominant *phoD* or *gcd* taxa with environmental factors (including pH, EC, SOM, SM, TN, TK, NH_4_-N, AK, NaHCO_3_-Pi, 0.1 M NaOH-Pi, HCl-Po, and 0.5 M NaOH-Pi). The results showed that 81.9.0 and 92.3% of the variations in the structures of *phoD* and *gcd* communities were explained in the first axis, and 12.8 and 6.8% were explained in the second axis, respectively (Figure 6). Figure 6 also shows that three MS and BSC samples are clustered across different groups, hence indicating clear differences in the compositions of *phoD* and *gcd* communities across different sites. The Monte Carlo permutation test indicated that pH, AK, and TK significantly affected the structures of *phoD* community (*p* < 0.05); while pH, SOM, 0.5 M NaOH-Pi, AK, TK, and SM were the main factors affecting *gcd* community.

## Discussion

### Soil Properties and P Speciation Responses to BSC Development

Revegetation on moving sand land facilitates the formation of BSC on semiarid sand land (Guo et al., 2008). The formation of BSC was affected by many environmental factors, including dust fall, hydrothermal condition, wind velocity, soil nutrient contents, vegetation type, and microbial activities (Li et al., 2002a; Zhao et al., 2010). Revegetation on mobile sand land can increase the roughness and decrease the surface albedo of the soil surface, thereby affecting wind velocity, heat balance, and surface water distribution (Li et al., 2002b). Meanwhile, plantation can protect surface soil from wind erosion and trap fine soil particles and dust that are rich in nutrients (Cao et al., 2008). The improvement of soil nutrient also increased the quantity and diversity of soil microbes (Zhang et al., 2019), including N-cycle- (Li et al., 2019a; Zhao et al., 2020b) and P-transformation-related taxa (Figure 2). The increase in soil AP along with BSC development was mainly due to the mineralization of OP, with IP dissolution merely accounting for a small proportion. This result can also be verified by the abundance of *phoD* and *gcd* genes. The increased quantity of soil *phoD* microbes in BSCs can improve ALP activity, which catalyzes the hydrolyzing reaction of OP to potentially plant-available orthophosphate (Cui et al., 2015). *gcd*-PSB may contribute less to the AP improvement of BSCs in semiarid sandy soil.

Fractions of AP increased over time, which mainly depended on the results of the *phoD*-harboring microbial function, although some of them were immobilized by microbial biomass (Cao et al., 2008). Atmospheric dust fall can increase soil AP, meanwhile, the functions of herbaceous plant roots may facilitate the liberation of P. NaHCO_3_-Po is a P speciation that can be easily mineralized (da Silva et al., 2017). In natural ecosystem soils, the absorbable AP for plants mainly originates from OP mineralization within SOM. This process is catalyzed by soil enzymes (e.g., ALP) mainly produced by relative microbes (Stewart and Tiessen, 1987). In this study, the easily mineralized OP (NaHCO_3_-Po) in BSCs were significantly higher than that in MS probably due to the increased SOM content and diversity of soil organisms, which also increased the amount of readily labile P (including H_2_O-Pi, NaHCO_3_-Pi, and NaHCO_3_-Po). The HCl-Pi (1.0 mol L^−1^, generally associated with Ca) and NaOH-Pi (0.1 mol L^−1^, generally associated with Fe and AL) fractions were considered moderate AP components (Hedley et al., 1982). Compared with MS, higher HCl-Pi, and NaOH-Pi were observed in BSCs probably due to the slight increase in pH, which can decrease the solubility of Ca-, Fe-, or Al-associated P and subsequently improve the extraction of fractions with NaOH or HCl (da Silva et al., 2017; Wang et al., 2021a). Given that P adsorption in arid or semiarid soils is generally slow, most of the retained or residual-P, which are insoluble in chemical extractors such as HCl, NaOH, H_2_O, or NaHCO_3_, also accounted for high proportions.

### ALP Activity and Abundance of *phoD* and *gcd* Genes

The formation and development of BSCs on surface soil inevitably alters soil microenvironments, thereby increasing the number of soil organisms (Song et al., 2020). Some studies have used *phoD* and/or *gcd* as major molecular markers of P-transformation microbes, and the variations in their abundance can represent the tendency of soil P-transformation (Chen et al., 2017; Luo et al., 2017; Li et al., 2019b; Hu et al., 2020). ALP is a key enzyme responsible for OP mineralization (Turner and Haygarth, 2005). In this study, ALP activity linearly increased along with BSC age, which was consistent with the variation trend of *phoD* gene abundance. This phenomenon can be attributed to the fact that the synthesis of ALP is regulated by the *phoD* gene and that the increased quantity of *phoD*-harboring microbes promotes the production of ALP (Tan et al., 2013). ALP activity can be influenced by many soil factors, among which SOM is the most important. A possible reason is that SOM provides carbon and nitrogen resources that increase microbial amount. Furthermore, ALP activity is affected by soil fraction because soil fractions may influence the function of distinct microbial communities due to their different mineralogical composition, adsorbed compounds on the surface, organic matter content, and microenvironments (Hemkemeyer et al., 2015; Luo et al., 2017). Similar to the *phoD* gene, the abundance of the *gcd* gene increased along with BSC development, but its value was much lower than that of *phoD*. Therefore, the contribution ratio of PSB to AP content may be very low in semiarid sandy soil.

### *phoD*- and *gcd*-Harboring Microbial Diversities and Community Structures

The α-diversity indices (including Shannon–Wiener, Chao 1, Pielou, and Simpson) of *phoD* and *gcd* communities all increased along with BSC age (Table 4). However, the species diversity of the *gcd* community was much lower than that of the *phoD* community. This result was consistent with the variations in the phosphorite dissolution rate and copies of the *gcd* gene, which also supported the contention that the availability of soil P mainly depends on the mineralization of OP in semiarid sandy soil. The formation of BSC on moving sand dunes facilitated a rapid increase in *phoD*- and *gcd*-harboring species. The number of observed species of the *phoD* or *gcd* community was the highest in BSC-20 soils among the four sites, where MS < BSC-8 < BSC-35 < BSC-20 (Table 4). This phenomenon highlights the structure dynamics of microbial community originating from secondary bare land, that is, these species rapidly increase due to improvements in soil environment at the early stage and then decrease due to intra- and inter-specific competitions at the stable stage (Cao et al., 2017). Therefore, similar to plant community succession, the developments of soil *phoD* and *gcd* microbial communities can be classified into the initial (0 a, MS), early (8 a), middle (20 a), and stable (35 a) stages of BSC (Supplementary Figures S1, S2).

Soil properties are direct factors that influence the structure of microbial communities. RDA results suggested that soil factors were significantly correlated to the structures of *phoD* and *gcd* communities (Figure 6). Meanwhile, BSC sites were significantly affected by pH and AK. Soil pH was reported as the primary driver of *phoD*-harboring community structure in arable soil (Wang et al., 2012; Ragot et al., 2015; Hu et al., 2018). Ragot et al. (2016) found that pH, organic carbon, and total nitrogen are important determinants responsible for the changes in *phoD* community structure at different P of grassland soil. Some studies reported that the application of K fertilizer can significantly alter the structure of soil microbial community, and available K is one of the key factors closely related to alterations in the composition of microbial community (Li et al., 2021; Xu et al., 2021). Wang et al. (2020) also observed that some soil factors including total K, organic carbon, total N, and available P played key roles in shaping the soil *phoD* microbial community. Most of the dominant *phoD* or *gcd* taxa were also affected by soil properties. However, not all *phoD*- or *gcd*-harboring taxa, e.g., the members of Gloeobacteraceae in the *phoD* community and the members of *Klebsiella* and *Serratia* in the *gcd* community, had important roles in the variations in P turnover function because of their low or stable abundance in BSCs (Chen et al., 2019).

In this study, the dominant *phoD*-harboring microbial phyla in all sites were Actinobacteria, Planctomycetes, and Proteobacteria, which were also reported in previous studies (Tan et al., 2013; Lagos et al., 2016; Luo et al., 2017). Meanwhile, the most dominant taxa in the *gcd* microbial community were Proteobacteria, *Rhizobium*, and *Planctomyces*. Despite differences in soil properties, the dominant *phoD*- and *gcd*-harboring microbes between MS and BSCs were similar and stable, thereby suggesting that most of the dominant phyla and genera in *phoD* and *gcd* communities were not changed by soil environmental variations (Ragot et al., 2015). Wang et al. (2021b) reported the compositions and their dynamics of soil *phoD* and *gcd* communities under a chronosequence of *C. microphylla* plantation in the same area of this study. We found that the dominant taxa and their variations of *phoD* and *gcd* communities in BSCs were similar to those in non-BSC soils, although their relative abundance were slightly different. Therefore, we infer that the basic compositions of *phoD* and *gcd* communities maybe mainly depend on local climate or soil type, because many highly resilient and resistant microbial subsets suitable for this environment widely exist in microbial communities (Suleiman et al., 2013). Although the basic compositions of *phoD* and *gcd* communities were not affected by BSC development, the dominant taxa quantitatively responded to the improvement in the soil properties of BSC. Therefore, the variations in the structures of *phoD* and *gcd* communities were mainly reflected in the waning and waxing relative abundance of dominant taxa similar to the observed distinctive taxa shown in Figures 4, 5. *Pseudomonas* was observed both in *phoD* and *gcd* communities as one of the dominant genera in MS and BSC sites, and its relative abundance remained stable along the BSC development. *Pseudomonas* promotes plant growth by contributing to the mineralization of OP or the solubilization of insoluble phosphate (Alori et al., 2017; Chen et al., 2019). *Rhizobium* and *Bradyrhizobium*, which had relatively high abundance in some sites, are free-living or symbiotic N_2_-fixers that have important roles in coupling P turnover and N cycling by affecting ALP activity and soil N pools (Luo et al., 2017). To further understand P turnover in soils, future studies should simultaneously investigate the microbes associated with C, N, and P cycling and the transcriptions or expressions of *phoD* and *gcd* genes.

## Conclusion

Soil nutrient content (SOM, TN, NH_4_-N, AP, and AK) and ALP activity gradually improved across the development of soil BSC under revegetated plantation on moving sand dunes. The abundance and diversities of *phoD* and *gcd* genes and the potential of OP mineralization linearly increased along with BSC age. *phoD*-harboring microbes were much more abundant than *gcd* in BSCs, thereby suggesting that an increase in soil AP across the BSC development mainly depended on OP mineralization, whereas the contribution ratio of the phosphate solubilizing function was very low in semiarid sandy soil. BSC development facilitated an increase in the availability of IP fractions, and NaHCO_3_-Pi and NaOH-Pi were positively correlated with the abundance of *phoD* and *gcd* genes and ALP activity.

In the *phoD* community, the phyla of Actinobacteria, Planctomycetes, and Proteobacteria and the family of *Streptomycetaceae* were identified as the most dominant taxa, Proteobacteria was the most dominant phylum, and *Rhizobium* and *Planctomyces* were the most dominant genera in the *gcd* community in BSC soils. The dominant taxa quantitatively responded to soil property improvement, while the basic compositions of dominant taxa did not change along with BSC development. Therefore, the structural dynamics of *phoD* and *gcd* communities were mainly reflected in the waning and waxing relative abundance of dominant taxa. Soil factors were significantly correlated to the structures of *phoD* and *gcd* communities, among which pH, AK, and TK were the same important factors.

## Data Availability Statement

The datasets presented in this study can be found in online repositories. The names of the repository/repositories and accession number(s) can be found in the article/**Supplementary Material**.

## Author Contributions

CC proposed the study, designed the experiments, analyzed data, participated in supervision, and wrote the manuscript. XZ and LP conducted experiments. YZ and ZC participated in experimental supervision and field investigation. All authors contributed to the article and approved the submitted version.

## Funding

This work was supported by the National Natural Science Foundation of China (No. 41877536).

## Conflict of Interest

The authors declare that the research was conducted in the absence of any commercial or financial relationships that could be construed as a potential conflict of interest.

## Publisher’s Note

All claims expressed in this article are solely those of the authors and do not necessarily represent those of their affiliated organizations, or those of the publisher, the editors and the reviewers. Any product that may be evaluated in this article, or claim that may be made by its manufacturer, is not guaranteed or endorsed by the publisher.

## Footnotes

^1^https://blast.ncbi.nlm.nih.gov/Blast.cgi

^2^ https://www.mothur.org

## Supplementary Material

Supplementary Table S1 Pearson correlations between marked taxa (relative abundances >1% in at least one sample) and soil properties.

Supplementary Figure S1 The rarefaction curves of high-throughput sequencing of *phoD* and *gcd* genes. MS, moving sand dune; BSC-8, 8-year-old biological soil crust; BSC-20, 20-year-old biological soil crust; and BSC-35, 35-year-old biological soil crust.

Supplementary Figure S2 Hierarchical clustering analysis based on the unweighted pair-group method across different *phoD* communities. MS, moving sand dune; BSC-8, 8-year-old biological soil crust; BSC-20, 20-year-old biological soil crust; and BSC-35, 35-year-old biological soil crust.

Supplementary Figure S3 Hierarchical clustering analysis based on the unweighted pair-group method across different *gcd* communities. MS, moving sand dune; BSC-8, 8-year-old biological soil crust; BSC-20, 20-year-old biological soil crust; and BSC-35, 35-year-old biological soil crust.
