# Supplementary material for: Dynamics of phoD- and gcd-Harboring Microbial Communities Across an Age Sequence of Biological Soil Crusts Under Sand-Fixation Plantation
Source: Front Microbiol. 2022 Mar 4;13:831888. doi: 10.3389/fmicb.2022.831888 (PMC8931599; doi:10.3389/fmicb.2022.831888)
Supplement: Supplementary Table S1 — Pearson correlations between marked taxa (relative abundances >1% in at least one sample) and soil properties. [file Table_1.DOCX]

Table S1 Pearson coefficients between marked taxa (relative abundances >1% in at least one sample) and soil properties

| Marked taxa | SM | pH | EC | SOM | TN | TP | NH_4_-N | AP | AK |
| --- | --- | --- | --- | --- | --- | --- | --- | --- | --- |
| *phoD*-harboring |  |  |  |  |  |  |  |  |  |
| Gemmataceae | -0.513 | -0.771 | -0.687 | -0.493 | -0.741 | -0.402 | -0.820 | -0.584 | -0.615 |
| Burkholderiales | -0.622 | -0.76 | -0.730** | -0.672* | -0.717** | -0.577* | -0.703* | -0.706* | -0.763** |
| Betaproteobacteria | -0.622* | -0.760** | -0.730** | -0.672* | -0.717** | -0.577* | -0.703* | -0.706* | -0.763** |
| Xanthomonadaceae | -0.644* | -0.867** | -0.889** | -0.903** | -0.896** | -0.634* | -0.772** | -0.950** | -0.870** |
| Sphingomonadaceae | 0.393 | 0.750** | 0.569 | 0.645* | 0.651* | 0.222 | 0.621* | 0.715** | 0.484 |
| Firmicutes | -0.017 | 0.527 | 0.144 | 0.214 | 0.27 | -0.243 | 0.491 | 0.393 | 0.073 |
| Paenibacillaceae | 0.041 | 0.562 | 0.189 | 0.253 | 0.312 | -0.213 | 0.52 | 0.434 | 0.115 |
| Rubrobacteraceae | 0.582* | 0.53 | 0.640* | 0.757** | 0.668* | 0.494 | 0.461 | 0.689* | 0.721** |
| *gcd*-harboring |  |  |  |  |  |  |  |  |  |
| Enterobacterales | -0.614* | -0.873** | -0.881** | -0.782** | -0.880** | -0.642* | -0.863** | -0.807** | -0.875** |
| Enterobacteriaceae | -0.606* | -0.864** | -0.877** | -0.781** | -0.873** | -0.636* | -0.852** | -0.801** | -0.873** |
| *Escherichia* | -0.593* | -0.851** | -0.867** | -0.767** | -0.854** | -0.638* | -0.831** | -0.789** | -0.862** |
| Sphingomonadaceae | -0.632* | -0.728** | -0.783** | -0.744** | -0.840** | -0.574 | -0.765** | -0.712** | -0.765** |
| Planctomycetales | 0.506 | 0.28 | 0.415 | 0.14 | 0.425 | 0.423 | 0.373 | 0.124 | 0.399 |
| Planctomyces | 0.496 | 0.301 | 0.429 | 0.168 | 0.441 | 0.448 | 0.389 | 0.137 | 0.412 |
| Planctomycetes | 0.492 | 0.287 | 0.413 | 0.145 | 0.423 | 0.411 | 0.384 | 0.129 | 0.402 |
| *Agrobacterium* | 0.603* | 0.649* | 0.594* | 0.358 | 0.666* | 0.405 | 0.710** | 0.449 | 0.555 |
| Alphaproteobacteria | 0.56 | 0.924** | 0.842** | 0.922** | 0.870** | 0.585* | 0.896** | 0.947** | 0.890** |
| *Rhizobium* | 0.538 | 0.928** | 0.862** | 0.945** | 0.891** | 0.673* | 0.882** | 0.966** | 0.883** |

Abbreviations: SM: soil moisture; EC: electrical conductivity; SOM: soil organic matter; TN: Total N; TP: total P; AP: available P; AK: available K; ALP: Alkaline phosphatase.

*: *P* < 0.05; **: *P* < 0.01.
