# Supplementary figures and images for: Dynamics of phoD- and gcd-Harboring Microbial Communities Across an Age Sequence of Biological Soil Crusts Under Sand-Fixation Plantation
Source: Front Microbiol. 2022 Mar 4;13:831888. doi: 10.3389/fmicb.2022.831888 (PMC8931599; doi:10.3389/fmicb.2022.831888)

observed\_species

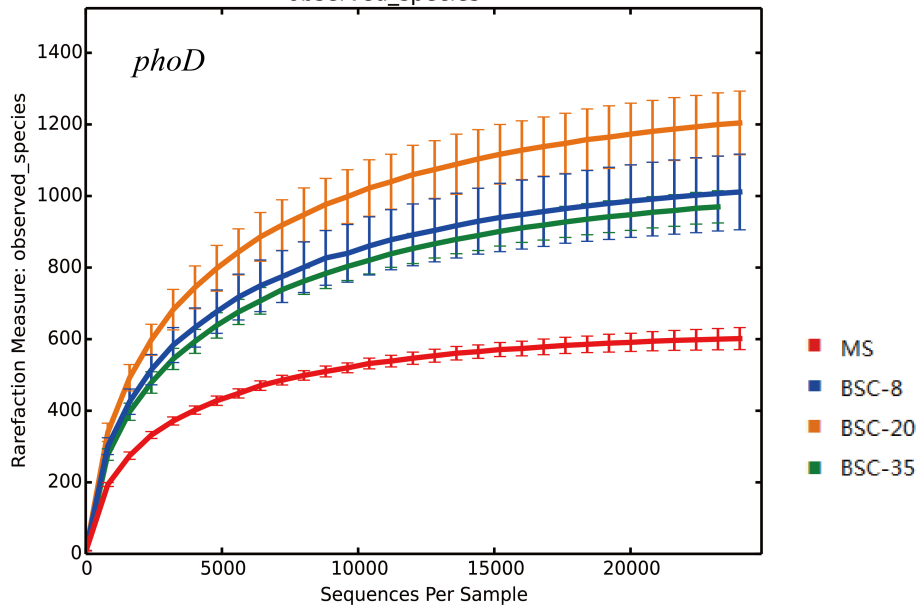

observed\_species

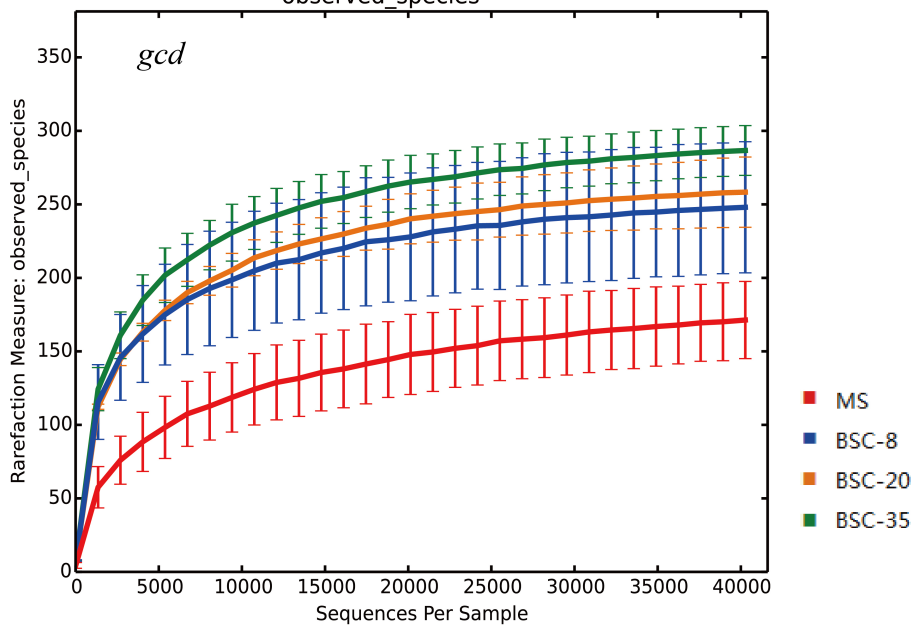

Supplement: Supplementary Figure S1 — The rarefaction curves of high-throughput sequencing of phoD and gcd genes. MS, moving sand dune; BSC-8, 8-year-old biological soil crust; BSC-20, 20-year-old biological soil crust; and BSC-35, 35-year-old biological soil crust. [file Data_Sheet_1.PDF]

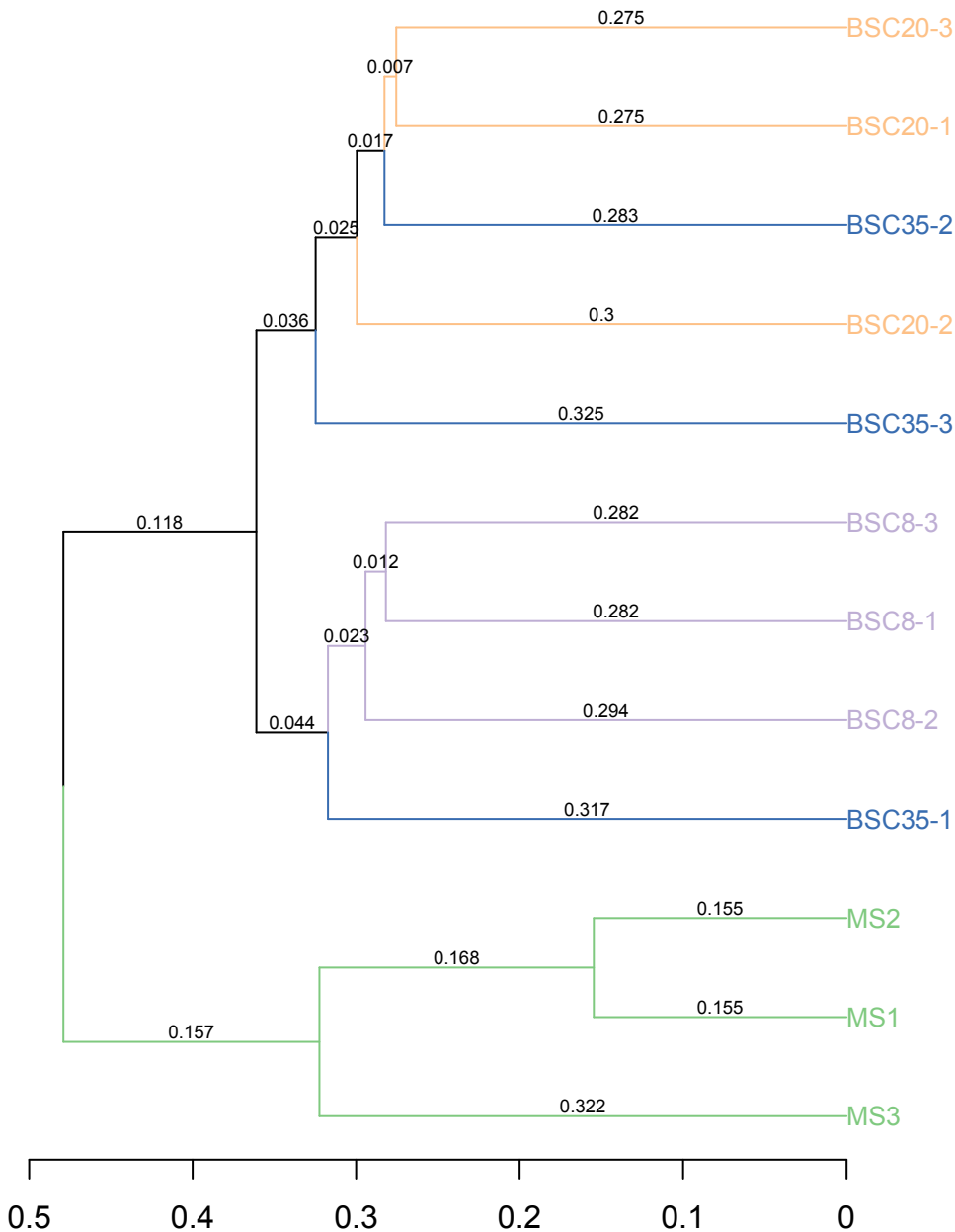

MS  
BSC8  
BSC20  
BSC35

BSC20-3

BSC20-1

BSC35-2

BSC20-2

BSC35-3

BSC8-3

BSC8-1

BSC8-2

BSC35-1

MS2

MS1

MS3

Supplement: Supplementary Figure S2 — Hierarchical clustering analysis based on the unweighted pair-group method across different phoD communities. MS, moving sand dune; BSC-8, 8-year-old biological soil crust; BSC-20, 20-year-old biological soil crust; and BSC-35, 35-year-old biological soil crust. [file Data_Sheet_2.PDF]

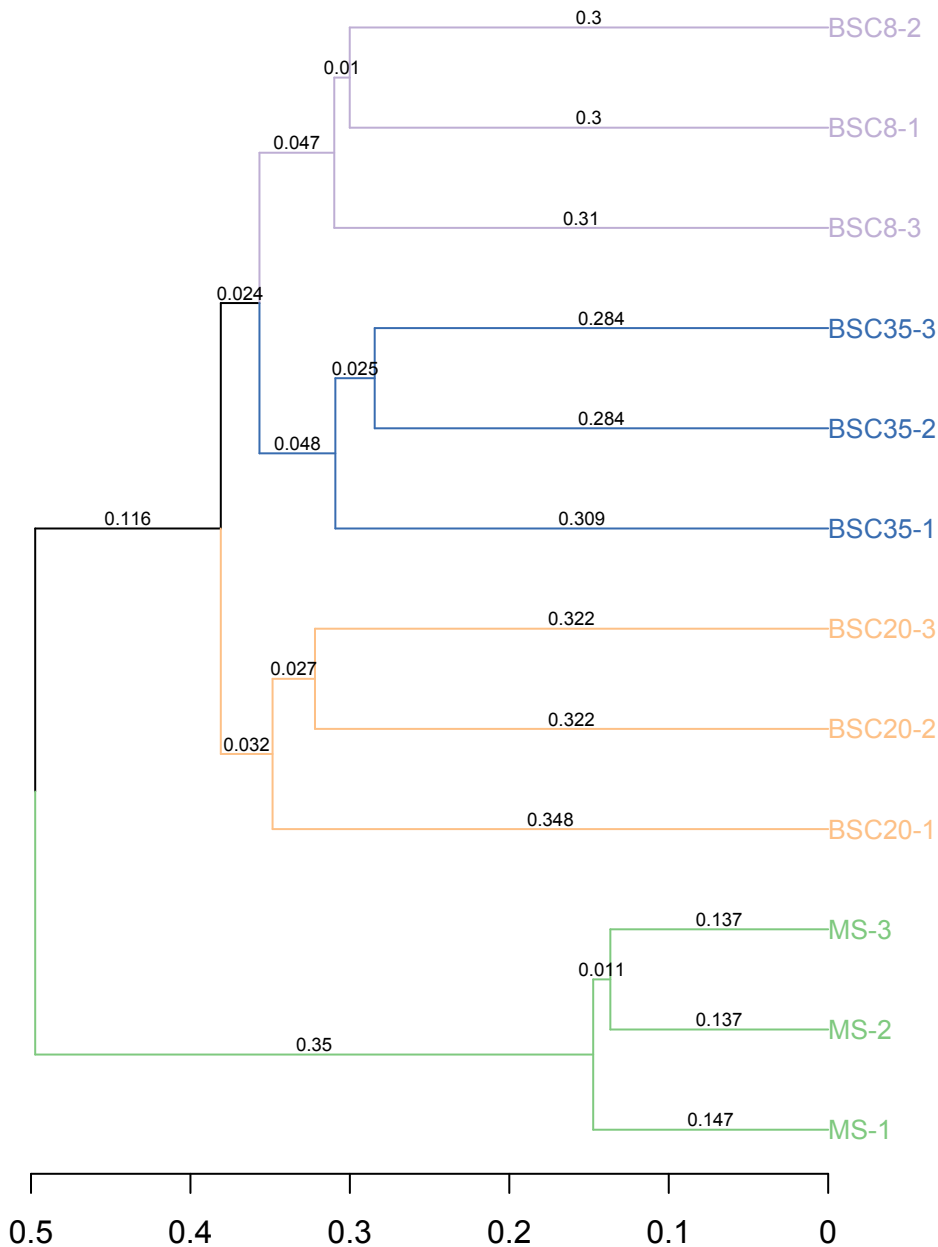

MS  
BSC8  
BSC20  
BSC35

Supplement: Supplementary Figure S3 — Hierarchical clustering analysis based on the unweighted pair-group method across different gcd communities. MS, moving sand dune; BSC-8, 8-year-old biological soil crust; BSC-20, 20-year-old biological soil crust; and BSC-35, 35-year-old biological soil crust. [file Data_Sheet_3.PDF]
